# Characterization of Phytochemicals in *Ulva intestinalis* L. and Their Action Against *SARS-CoV-2* Spike Glycoprotein Receptor-Binding Domain

**DOI:** 10.3389/fchem.2021.735768

**Published:** 2021-09-27

**Authors:** Seema A. Kulkarni, Sabari B.B. Krishnan, Bavya Chandrasekhar, Kaushani Banerjee, Honglae Sohn, Thirumurthy Madhavan

**Affiliations:** ^1^ Department of Biotechnology, School of Bioengineering, SRM Institute of Science and Technology, Chengalpattu, India; ^2^ Computational Biology Laboratory, Department of Genetic Engineering, School of Bioengineering, SRM Institute of Science and Technology, Chengalpattu, India; ^3^ Department of Chemistry and Department of Carbon Materials, Chosun University, Gwangju, South Korea

**Keywords:** SARS-CoV-2 spike S1 subunit, Ulva intestinalis L., phytochemicals, GC-MS, COVID-19, molecular docking, ADMET studies, conceptual DFT

## Abstract

*Coronavirus* disease-2019 (COVID-19) has caused a severe impact on almost all aspects of human life and economic development. Numerous studies are being conducted to find novel therapeutic strategies to overcome COVID-19 pandemic in a much effective way. *Ulva intestinalis* L. (*Ui*), a marine microalga, known for its antiviral property, was considered for this study to determine the antiviral efficacy against severe acute respiratory syndrome-associated *Coronavirus*-2 (*SARS-CoV-2*). The algal sample was dried and subjected to ethanolic extraction, followed by purification and analysis using gas chromatography-coupled mass spectrometry (GC-MS). Forty-three known compounds were identified and docked against the S_1_ receptor binding domain (RBD) of the spike (S) glycoprotein. The compounds that exhibited high binding affinity to the RBD of S_1_ protein were further analyzed for their chemical behaviour using conceptual density-functional theory (C-DFT). Finally, pharmacokinetic properties and drug-likeliness studies were carried out to test if the compounds qualified as potential leads. The results indicated that mainly phenols, polyenes, phytosteroids, and aliphatic compounds from the extract, such as 2,4-di-tert-butylphenol (2,4-DtBP), doconexent, 4,8,13-duvatriene-1,3-diol (DTD), retinoyl-β-glucuronide 6′,3′-lactone (RBGUL), and retinal, showed better binding affinity to the target. Pharmacokinetic validation narrowed the list to 2,4-DtBP, retinal and RBGUL as the possible antiviral candidates that could inhibit the viral spike protein effectively.

## Introduction

COVID-19, a contagious viral disease caused by *SARS-CoV-2*, was declared as a public health emergency of international concern by the World Health Organization (WHO) on 30 January 2020, and as a pandemic on March 11, 2020 (Ge et al., 2020). According to the recent pandemic situation report released by the WHO, *SARS-CoV-2* has infected nearly 180 million individuals, causing about four million deaths. Being a positive, single-stranded RNA virus of size 50–200 nm and genome size of 29.9 k ribonucleotides, it is the most recent member included in the *Betacoronavirus* genus of the *Orthocoranavirinae* subfamily of coronaviruses (Lu et al., 2020). The viral genome was found to encode twelve main proteins, of which two, the spike glycoprotein and the main protease (M^pro^) have gained attention as potential COVID-19 drug targets (Pavlova et al., 2021). The availability of structural details of these two proteins has accelerated computational studies. The thermodynamically favoured irreversible inhibition of M^pro^ by Michael acceptors has been studied by computational methods such as molecular dynamics and density functional theory (Poater 2020; Ramos-Guzmán et al., 2021; Zanetti-Polzi et al., 2021). The covalent and non-covalent binding free energies of M^pro^inhibitors have been studied to aid in rational drug discovery and design for targeted antiviral therapy (Awoonor-Williams and Abu-Saleh, 2021). Several experimentations suggest that *SARS-CoV* and *SARS-CoV–2* have a sequence identity of approximately 79 percent, and both variants use angiotensin converting enzyme 2 (ACE2) as their cellular receptor. Similarly, some studies suggest that the infectivity rate varies with amino acid change in the spike protein, and the adsorption of S protein on gold nanoparticles was completely dependant on the size of the core nano-gold (Bette et al., 2021; Yokoyama and Ichiki, 2021). The spike glycoprotein is comprised of two subunits, the S_1_, which has the receptor binding domain, and the S_2_, which facilitates membrane fusion and endocytosis of the virus (Walls et al., 2020). Several studies have shown that *SARS-CoV-2* utilizes the S_1_ protein to bind to the functional receptor human ACE2 (hACE2) at the RBD. The same mechanism was used for viral entry by *SARS-CoV* too. Eventually S_2_ protein aids in fusion of viral particles in the host. The receptor-binding motif (RBM) in RBD is the main functional motif and is composed of two regions (region 1 and region 2) that form the interface between the S protein and hACE2. The region outside the RBM in RBD also plays an important role in maintaining the structural stability of the RBD (Li et al., 2003; Yi et al., 2020; Zhou et al., 2020).

The current challenge faced by the health sector is the resistance and insensitivity of the virus to existing drugs, and those drugs that have an edge over the virus were found to have some detrimental side effects. Drugs such as hydroxychloroquine and chloroquine (FDA-approved drugs that are effective against malaria, lupus, and rheumatoid arthritis) were found to hamper this viral infection, but the risks of developing cardiovascular and renal disorders were found in many of its consumers (FDA, 2020). Also, the recovery rate fluctuated from region to region, in fact, from person to person, with varying degrees of side-effects, forcing the WHO to halt the solidarity trial of hydroxychloroquine a few months after the COVID-19 outbreak.

*In silico* techniques play an important role in accelerating research to identify potential leads against *SARS-CoV-*2. Molecular docking, molecular dynamic simulation and drug repurposing are the strategies currently practiced for drug development against COVID-19 (Acharya et al., 2020). Molecular dynamic simulation studies futher help to substantiate the reciprocity between the protein and the ligand. Such tools can be exploited for drug developmental studies which further aid in lead optimization with increased specificity and selectivity (Raudah et al., 2020). Various herbs and plant-based compounds are being tested for possible antiviral activity against *SARS-CoV-2* (Anand et al., 2021). *Ui*, also called gutweed or grass kelp, a common but often unnoticed macro alga, was mainly studied for its anti-microbial and anti-cancer properties *in vitro*, however, few studies were published on its anti-viral activity (Morán-Santibañez et al., 2016; Klongklaew et al., 2020). It is a member of the *Ulvaceae* family, which belongs to the Chlorophyta (green seaweed) division (Class: Ulvophyceae, Order: Ulvales). It is found to be a euryhaline and thus can grow even in freshwaters, exclusively in nutrient-rich niches such as in water bodies that receive industrial and farm discharges, and low tidal zones. These tubular algae can reach up to 0.3 m in length, with a thickness of about 0.02 m, and exhibit a perennial isomorphic biphasic reproductive cycle. Considering its abundance in the Coromandel coastline of South India, and its possible action against viruses such as the measles *Morbillivirus*in Vero cell lines (Morán-Santibañez et al., 2016), *Ui* was considered as the source of phytochemicals that can serve as possible lead compounds against the S protein RBD of *SARS-CoV-2*.

## Materials and Methods

### Sample Collection and Preparation

**FIGURE 1:**
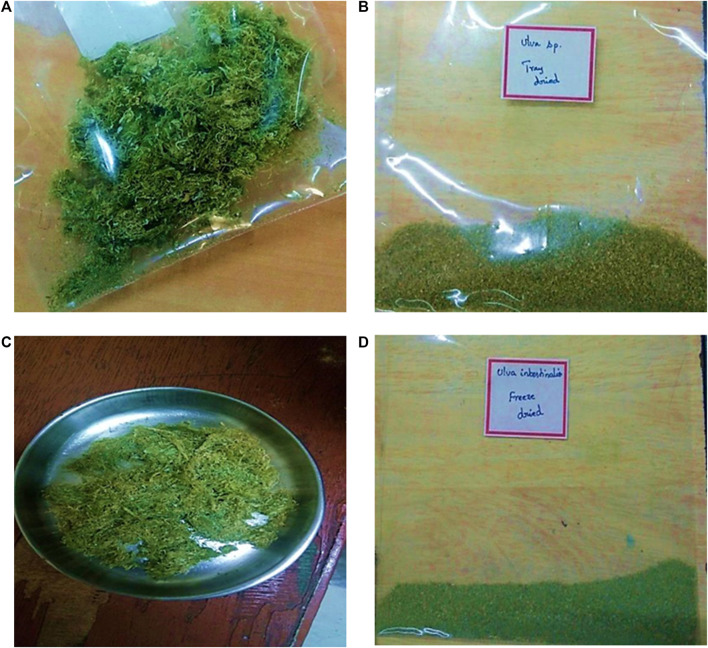
Samples of *U. intestinalis*used for Soxhlet extraction using ethanol. **(A)** Tray-dried sample **(B)** powdered form of tray-dried sample. **(C)** Freeze-dried sample **(D)** powdered form of freeze-dried sample.

The alga *Ui* were collected from the Olaikuda area (Gulf of Mannar) situated near North Mandapam, Rameswaram, Tamil Nadu, India, with the help of the Central Marine Fisheries Research Institute, Mandapam, and Rajendra Kumar Algae Project Center, Mandapam. The algal sample was washed thoroughly with water to remove dirt and debris and packed safely in polythene zip-lock bags. Upon reaching the laboratory it was dried using a tray drier (Figures 1A,C), mainly to concentrate the extract, preserve the hydrolabile compounds, and prevent the growth of bacteria and mold.

### Isolation and Identification of Phytochemicals

Phytochemical extraction was performed by Soxhlet extraction. The dried sample (∼60 g) was pulverized using a mortar and pestle (Figures 1B,D), and transferred into a thimble in the extraction tube. The extraction solvent used was 95% ethanol (100 ml). The all-glass Soxhlet apparatus was set up according to the standard protocol and was run for 6 h at 78°C using an isomantle. The extract was analyzed for the phytochemicals using a 7890B GC coupled with a 5977A mass selective detector (MSD). The chromatographic column used for GC was HP-5MS of dimensions 30 m × 250 μm × 0.25 μm (length, inner diameter, and film thickness, respectively). It is a bonded, cross-linked, and solvent-rinsable non-polar column made of (5%-phenyl)-methylpolysiloxane, with a capillary tubing made of fused silica (Agilent Technologies, Santa Clara, CA). The volume of the sample injected was 1 μl and the flow rate of the carrier gas (helium) was 1.0 ml.min^−1^ with a split ratio of 1:1. The injection port temperature was 250°C. The system started with a 2 min-hold at 50°C, then ramped 3°C per minute until the temperature reached 270°C. The system was on hold at this temperature for 20 min. Simultaneously, the separated samples were fed automatically to the MSD at an interface temperature of 280°C. The electron ionization was performed at 70 eV, and the scan range of the system was 40–700 m/*z*. The total run time of the process was 95 min. The retention indices of the compounds were determined relative to trichloromethane, the standard compound selected for data analysis. Further, the compounds were identified by comparing their mass spectra with the data in NIST-14 Mass Spectral Data Library.

### Preparation of Ligands and Target

The three-dimensional chemical structures of the identified phytochemicals were obtained from PubChem (https://pubchem.ncbi.nlm.nih.gov/). These were then saved as SDF files. The energy minimization and format conversion of these structures were performed in PyRx software (Dallakyan and Olson 2015). The default energy minimization parameters were the universal force field and the conjugate gradient algorithm. Once energy minimization was completed, the structures were rewritten as PDBQT files. The target protein used in this study was S_1_ receptor binding domain of the spike (S) glycoprotein. The three-dimensional structure of RBD was retrieved from a complex of ACE2 and RBD (PDB ID: 6M0J) from the Protein Data Bank (RCSB-PDB; https://www.rcsb.org/). As the first step, the optimization of protein structures was performed using AutoDock Tools by deleting chain A, water molecules, and co-crystal ligands. The missing atoms were then repaired, and polar hydrogens were added. Charges were distributed and minimized over the protein structure. The structure was then saved in PDBQT format.

### Active Site Prediction and Grid Box Parameters

**FIGURE 2:**
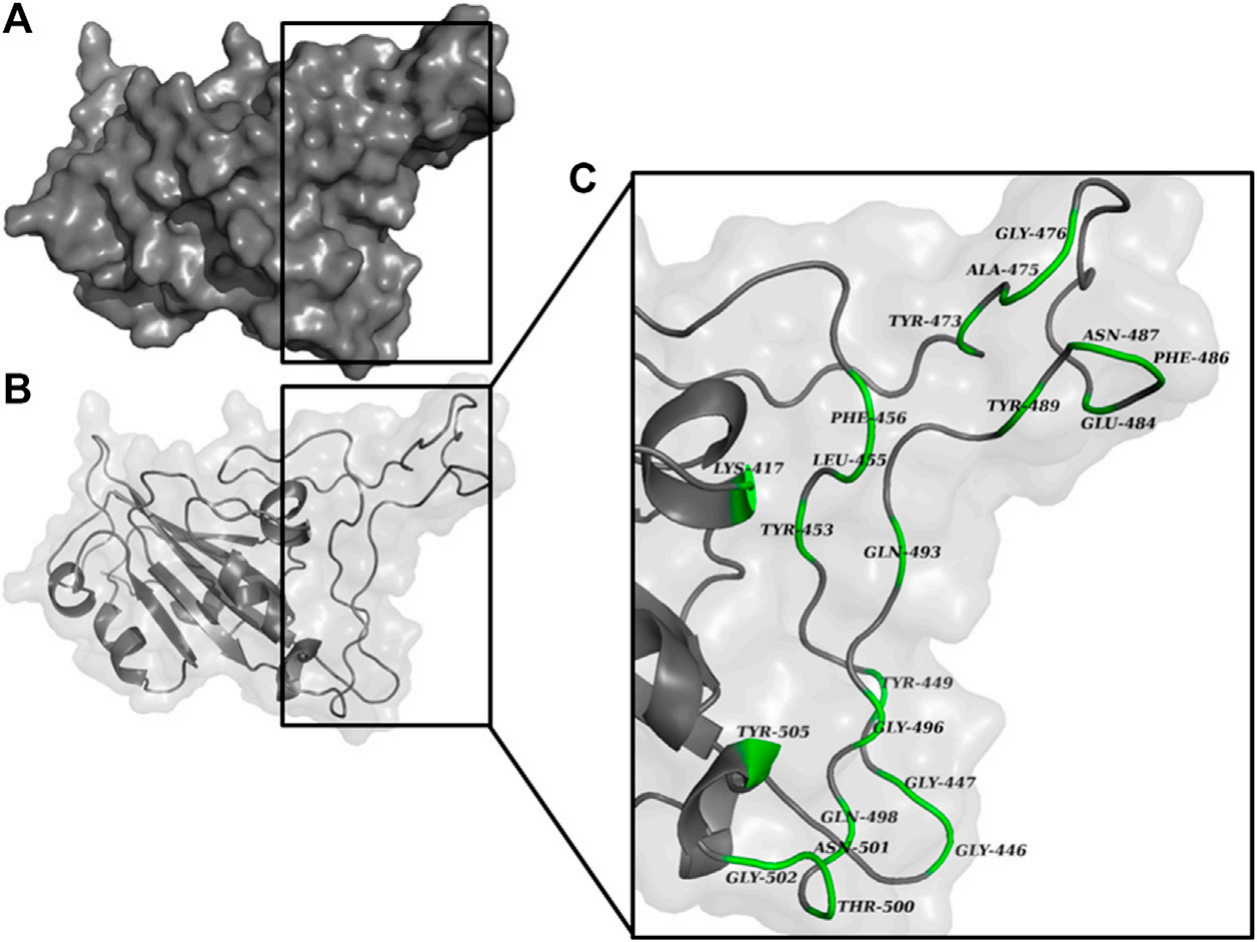
RBD of S_1_ protein represented as **(A)** surface, and **(B)** chain. The magnified view of the RBD **(C)** shows the possible interacting residues (green) in <5.0 Å vicinity with ACE2.

An active site is defined as a groove or pocket of an enzymatic or non-enzymatic protein which facilitates ligand binding or biochemical reactions (Pravda et al., 2014). The characteristics of the active site are mainly determined by the active site residues (Srinivasan, 2020), and various studies have characterized the possible active site residues of RBD of S_1_ subunit of spike protein (Figure 2). Tyr449, Tyr453, Arg454, Lys458, Ser459, Ser469, Glu471, Phe486, Asn487, Tyr489, Leu492, Gln493, Gly496, Gln498, Thr500, Asn501, Gly502, and Tyr505 were the reported active site residues (Lan et al., 2020; Kulkarni et al., 2020; Prajapat et al., 2020). These residues were further validated using the ‘Zone’ function in UCSF Chimera software (https://www.cgl.ucsf.edu/chimera/). The zone parameter was set to “<5.0 Å from currently selected atoms” (Ashraf et al., 2014), where the currently selected atoms were the atoms of chain A. The mean of the X, Y, and Z coordinates of the final atom of each interacting residue highlighted by UCSF Chimera was calculated and applied as the dimension of the grid-box center. The grid size was manually adjusted to cover the interacting residues. Further, the values of these coordinates were saved as a configuration text file which was later used for docking.

### Molecular Docking and Target-Ligand Visualization

Molecular docking is an *in silico* approach which is used to predict the conformational binding energy of ligands to a preferred target using matching and scoring algorithms (Leach et al., 2006). In this experiment, we have used AutoDockVina (Trott and Olson, 2010) in PyRx software as the docking tool, The optimal binding energy of the ligands was obtained based on least root mean square deviation (RMSD) for each conformers of a particular ligand, and arranged in ascending order to select the best ligand(s) for further calculating the chemical behaviour using C-DFT and pharmacokinetic analyses. PyMOL (https://pymol.org/), an open-source molecular visualization software was used to identify the polar contacts (H-bonds) between the ligand and the interacting active site residue, and develop printable figures of this interaction. To analyze hydrophobic interactions between the ligand and residues, another visualization software, BIOVIA Discovery Studio Client 2020 (https://discover.3ds.com/discovery-studio-visualizer-download) was used.

### Conceptual DFT Analysis

Conceptual Density-functional theory (C-DFT) is a computational method to predict chemical behaviour of the compounds (Poater et al., 2010; Domingo et al., 2016). Density-functional theory(DFT) has been developed from Hohenberg-Kohn theorem, which is an *in-silico* quantum mechanical modeling strategy used to determine the properties of a many-electron systems, using spatially-dependent electron density functionals (Hohenberg and Kohn, 1964; Kohn and Sham, 1965). C-DFT, a sub-field of DFT, helps to analyze the molecular orbital energies of conformers and can give rise to cues for understanding the structure-activity relationship of the molecule (Parr and Yang, 1989; Geerlings et al., 2003; Sarkar and Chattaraj, 2021a; Sarkar and Chattaraj, 2021b). To describe the orbital properties of a molecule, ten different molecular descriptors, known as the global reactivity descriptors and its derivatives, were considered *viz.* total energy (E_γ_; in eV), molecular dipole moment (D_p_; in Debye units), the energy of the lowest unoccupied molecular orbit (LUMO) (E_LUMO_; in eV), the energy of the highest occupied molecular orbit (HOMO) (E_HOMO_; in eV), energy gap (ΔE; in eV), absolute hardness (η; in eV), global softness (σ; in eV^−1^), electronegativity (χ), chemical potential (μ; in eV), and global electrophilicity index (ψ; in eV^−1^) (Chattaraj et al., 2003; Chattaraj et al., 2006). These molecular descriptors are calculated based on the electron density of molecules using Fukui’s molecular orbital theory (Fukui 1982; Ayers and Parr, 2000). E_LUMO_ and E_HOMO_ are the primary and the most important descriptors which determine the ability of a molecule to accept or donate electrons. D_p_ is the measure of the total polarity of a system. It is also a positive indicator of the reactivity of the molecule. It was found that the higher the D_p_, the greater the reactivity of the molecule (Roy et al., 2006; Mert et al., 2011). The derived descriptors of E_LUMO_ and E_HOMO_ are ΔE, η, σ, χ, μ, and ψ, which also account for the ability of the molecule to interact and contribute to electron sharing or transfer with the target by transiting from HOMO to LUMO. For example, if ΔE is found to be less, the molecule can easily transit from HOMO to LUMO (Chattaraj and Roy, 2007; Bostan et al., 2012). It represents the chemical reactivity and kinetic stability of the molecule; if χ is found to be less, the inhibitory effect of the ligand is higher (Zhan et al., 2003). As the first step in determining these descriptors, the selected ligands were optimized using the Becke-3-parameter, Lee-Yang-Parr (B3LYP) function (Becke 1988; Lee et al., 1988) with 6-311G(2d, p) basis set in Gaussian-16 software (http://gaussian.com/gaussian16/) (Frisch et al., 2016). B3LYP is the most popular functional used in molecular quantum mechanical modeling and is derived from a defined set of atomic/molecular energies and potentials.

### Pharmacokinetic and Drug-Likeliness Analyses

The drug-likeliness and pharmacokinetic properties such as Absorption, Distribution, Metabolism, Excretion, and Toxicity (ADMET) of the selected ligands were predicted. The Drug Likeliness Tool (DruLiTo; http://www.niper.gov.in/pi_dev_tools/DruLiToWeb/DruLiTo_index.html), an open-source drug-likeness software developed by the Department of Pharmacoinformatics, National Institute of Pharmaceutical Education and Research (NIPER), Punjab, India was used to analyze drug likeliness by checking whether the ligands violate any of Lipinski’s Rule of Five (RO5), or would pass the Ghose and Veber filters. A reliable online tool for pharmacokinetic predictions of small molecules, pkCSM (http://biosig.unimelb.edu.au/pkcsm/), was used to predict the ADMET properties of the ligands (Pires et al., 2015), in which the canonical or isomeric SMILES of the ligands from Pub Chem were given as input.

## Results

### Chemical Composition of Extract

**FIGURE 3:**
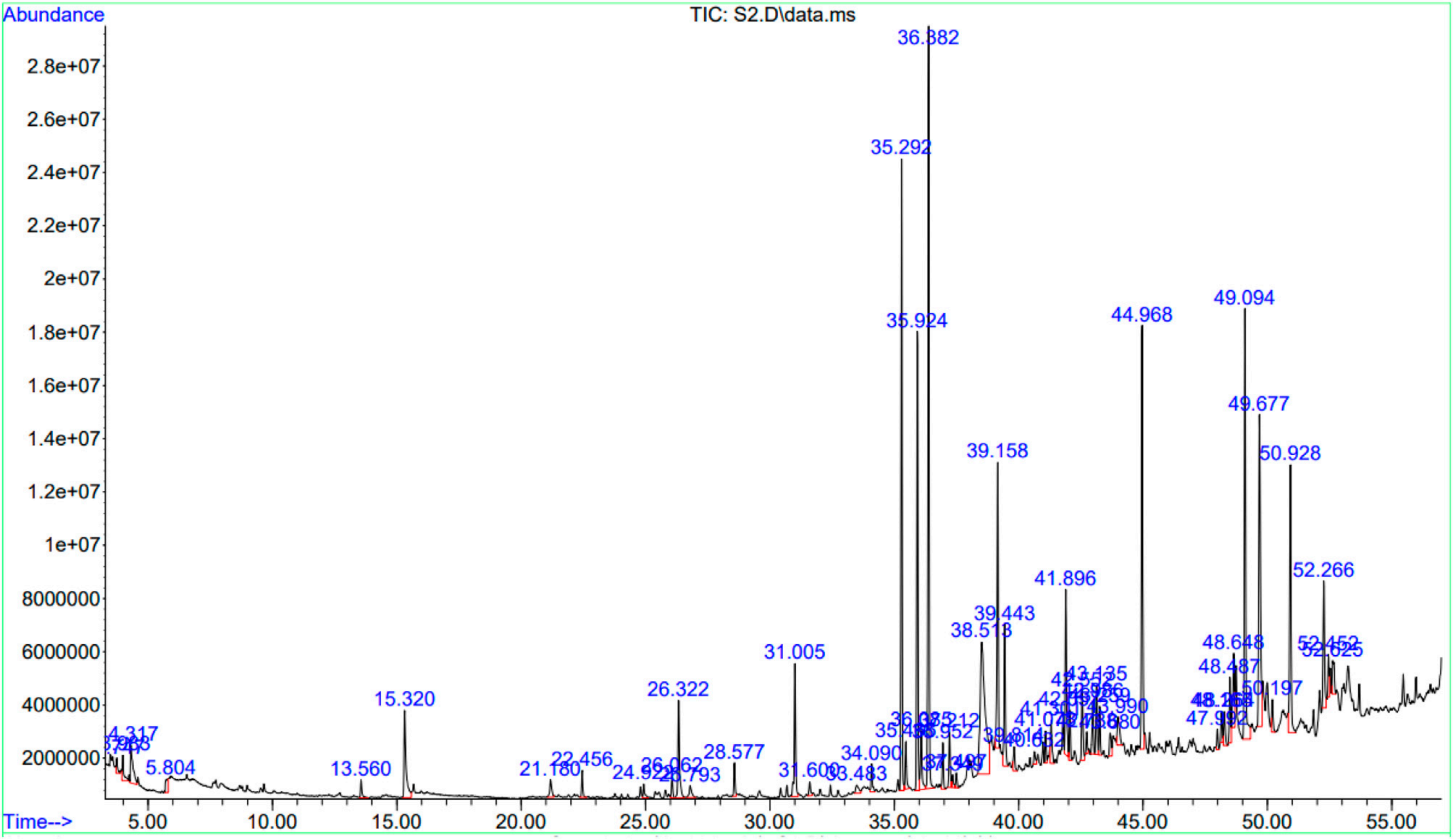
Chromatogram showing the results of GC-MS. The chromatogram was plotted against retention time in minutes (X-axis), and signal abundance (Y-axis). The collected fractions were fed automatically into an MS.

The GC-MS data of the *Ui* ethanolic extract showed 55 peaks (Figure 3), and on comparison with NIST-14 library, 43 known phytochemicals were identified (Table 1). The phytochemical class analysis revealed that 18 phytochemicals were simple carboxylic acids, fatty acids, or their derivatives (palmitic acid, HIP, methylpalmitate, ethylpalmitate, butanoic acid, paullinic acid, doconexent, allyl stearate, ethyllinolelaidate, ethyllinolenate, ethylelaidate, icosapent, MHDTE, BOD4E, BOD3E, BTES, DPPP, and propyllinoleate), seven belonged to terpenoid class (damascene, cyclosativene, dihydroactinolide, 3-DOCH, phytol, CMBA, and oxymesterone), three (6.98%) each were aldehydes and its derivatives (methylglyoxal, furfural, and retinal), alcohols and its derivatives (TAA, 1-heptatriacotanol, and DTD), alkene hydrocarbons and its derivatives (cetene, 8-heptadecene, and 9-octadecene), and alkane hydrocarbons and its derivatives (myristyl chloride, TMHA, and EEBOD), two were monoglycerides (2-monopalmitin and 1-monolinolein), and one each were an organosulfur compound (DMSO), an aromatic hydrocarbon (azulene), a phenol (2,4-DtBP), and a glycoside (RBGUL). The peak corresponding to HIP showed the highest signal abundance of >2.8×10^7^, however, the mean relative peak area of phytol (21.404%) was found to be the widest, followed by 2-monopalmitin, 9-octadecene, palmitic acid, and other compounds. The details of the GC-MS analysis such as peak number(s), retention time(s), and mean relative peak area are presented in Table 1.

**TABLE 1:** GC-MS data of the phytochemicals present in *Ui* extract.

Peak No(s)	Retention time(s) (min)	Compound	a_Peak Area_ (%)
1	3.747	Methylglyoxal	0.237
3	4.317	Furfural	1.110
4	5.804	DMSO	0.445
5	13.560	TAA	0.327
6	15.320	Azulene	2.135
7	21.180	Damascone	0.422
8, 13	22.456, 28.577	Cetene	0.877
9	24.922	Myristyl chloride	0.253
10	26.062	Cyclosativene	0.342
11	26.322	2,4-DtBP	1.902
12	26.793	Dihydroactinolide	0.355
14	31.005	8-Heptadecene	2.164
15	31.600	3-DOCH	0.265
16, 27	33.483, 38.513	Palmitic acid	8.207
17, 18	34.090, 35.292	9-Octadecene	9.082
19	35.465	TMHA	0.761
20, 22, 23, 33, 34	35.924, 36.382, 36.952, 41.301, 41.896	Phytol	21.404
21	36.085	HIP	1.134
24	37.212	CMBA	0.904
25, 41	37.349, 43.680	1-Heptatriacotanol	0.740
26	37.497	Methylpalmitate	0.237
28	39.158	Ethylpalmitate	4.206
29	39.443	Butanoic acid	2.250
30	39.814	Paullinic acid	0.428
31, 37	40.632, 42.738	Doconexent	0.524
32	41.078	Allyl stearate	0.445
35	42.057	DTD	0.734
36	42.552	Retinal	1.201
38	42.986	Ethyllinolelaidate	0.866
39	43.135	Ethyllinolenate	1.095
40	43.259	Ethylelaidate	0.658
42	43.990	Icosapent	0.782
43, 50	44.968, 49.677	2-Monopalmitin	13.139
44	47.992	EEBOD	0.293
45	48.165	MHDTE	0.447
46	48.264	BOD4E	0.606
47	48.487	1-Monolinolein	1.059
48, 55	48.648, 52.625	BOD3E	2.347
49	49.094	BTES	7.174
51	50.197	RBGUL	0.487
52	50.928	DPPP	4.517
53	52.266	Oxymesterone	2.362
54	52.452	Propyllinoleate	0.619

a_Values_ indicate the mean relative peak area. For compounds identified with more than one retention time, this value was presented to be a summation of the individual mean relative peak areas.

DMSO: Dimethyl sulfoxide; TAA: Tert-amyl alcohol; 2,4-DtBP: 2,4-Di-tert-butylphenol; 3-DOCH: 3-(6,6-Dimethyl-5-oxohept-2-enyl)cycloheptanone; TMHA: 3,7,11,15-Tetramethylhexadecylacetate; HIP: Hept-3-yl isobutyl ester of phthalic acid; CMBA: Cholestan-3-ol, 2-methylene- (3β,5α)-; DTD: 4,8,13-Duvatriene-1,3-diol; EEBOD: 3-Ethyl-5-(2-ethylbutyl)octadecane; MHDTE: Methyl 4,7,10,13-hexadecatetraenoate; BOD4E: Butyl 6,9,12,15-octadecatetraenoate; BOD3E: Butyl 9,12,15-octadecatrienoate; BTES: But-3-enyl tridecyl ester of sebacic acid; RBGUL: Retinoyl-β-glucuronide 6′,3′-lactone; DPPP: Di-n-2-propylpentylphthalate.

### Molecular Docking

**FIGURE 4:**
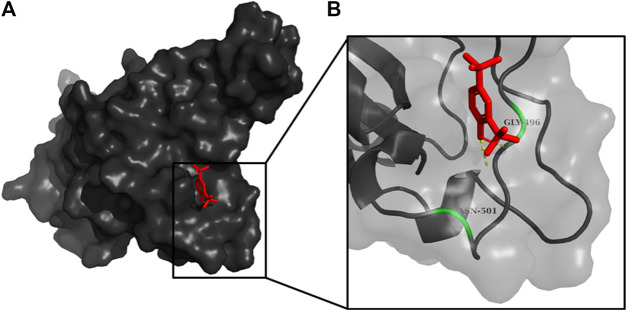
**(A)** The binding pose of 2,4-DtBP (red) in RBD of S1 protein. **(B)** The hydrogen bonds (yellow) formed between 2,4-DtBP and the interacting residues, Gly496 and Asn501 are also shown.

**FIGURE 5:**
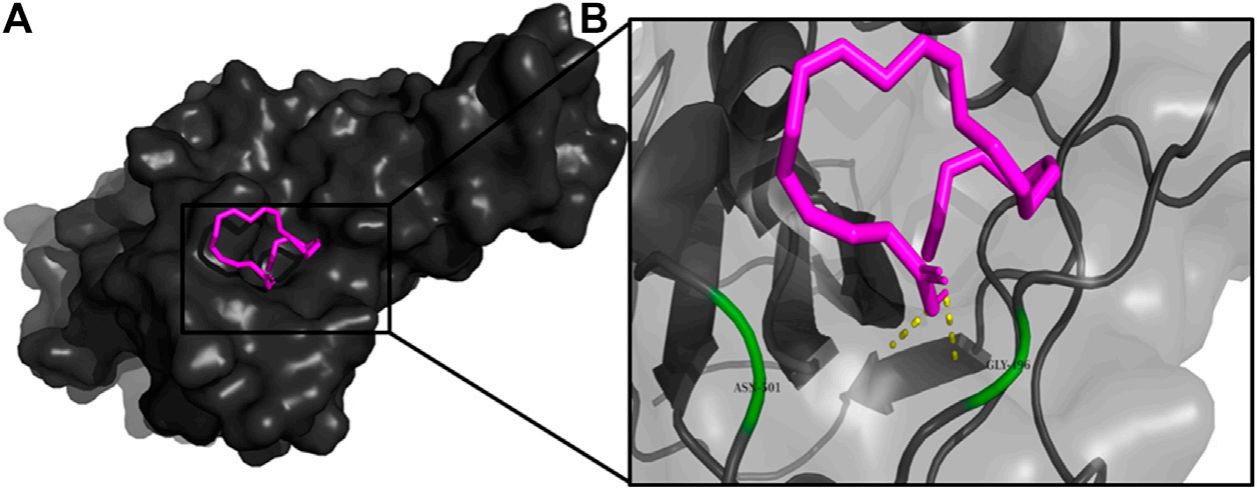
**(A)** The binding pose of doconexent (magenta) in RBD of S1 protein. **(B)** The hydrogen bonds (yellow) formed between doconexent and the interacting residues, Gly496 and Asn501 are also shown.

**FIGURE 6:**
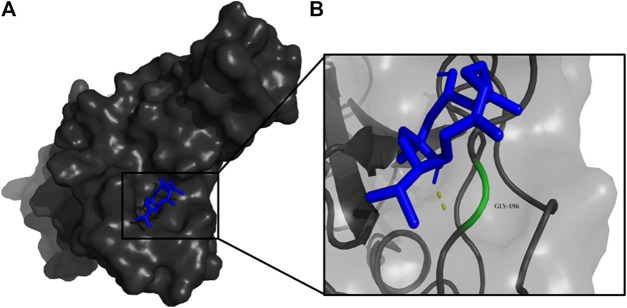
**(A)** The binding pose of DTD (blue) in RBD of S1 protein. **(B)** The hydrogen bond (yellow) formed between DTD and the interacting residue, Gly496, is also shown.

**FIGURE 7:**
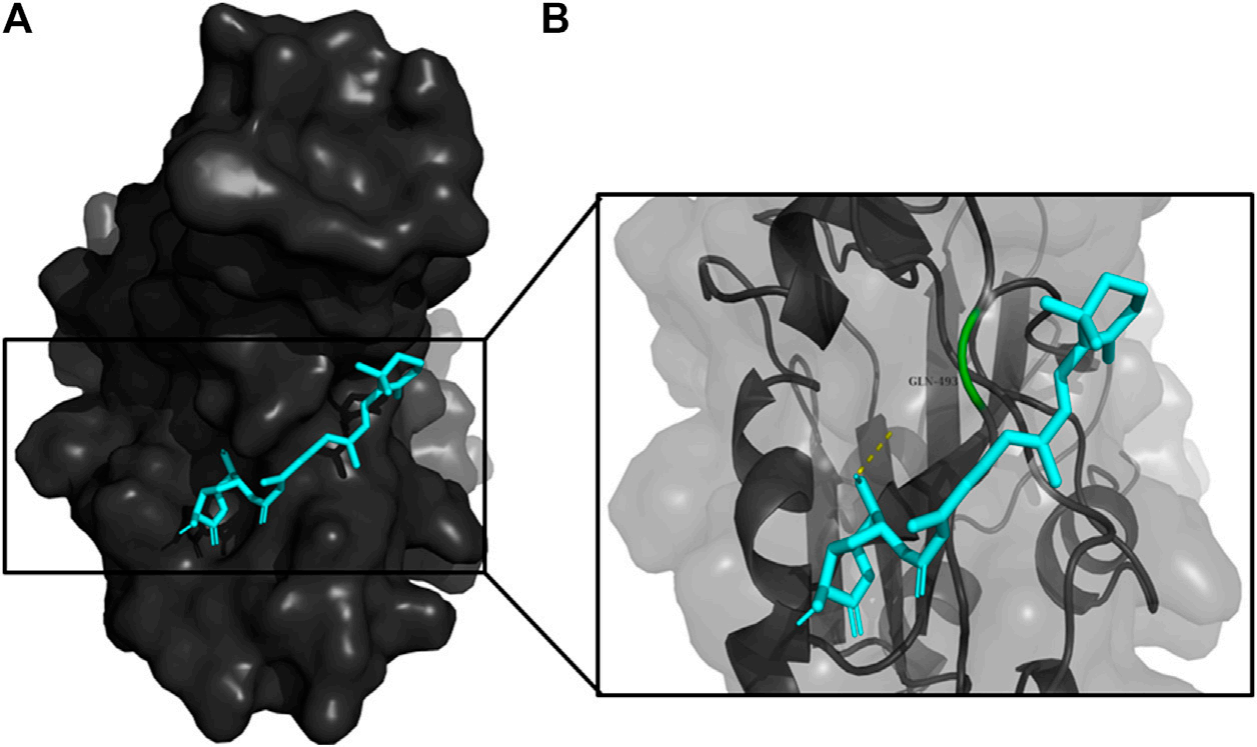
**(A)** The binding pose of RBGUL (cyan) in RBD of S1 protein. **(B)** The hydrogen bond (yellow) formed between RBGUL and the interacting residue, Gly493, is also shown.

**FIGURE 8:**
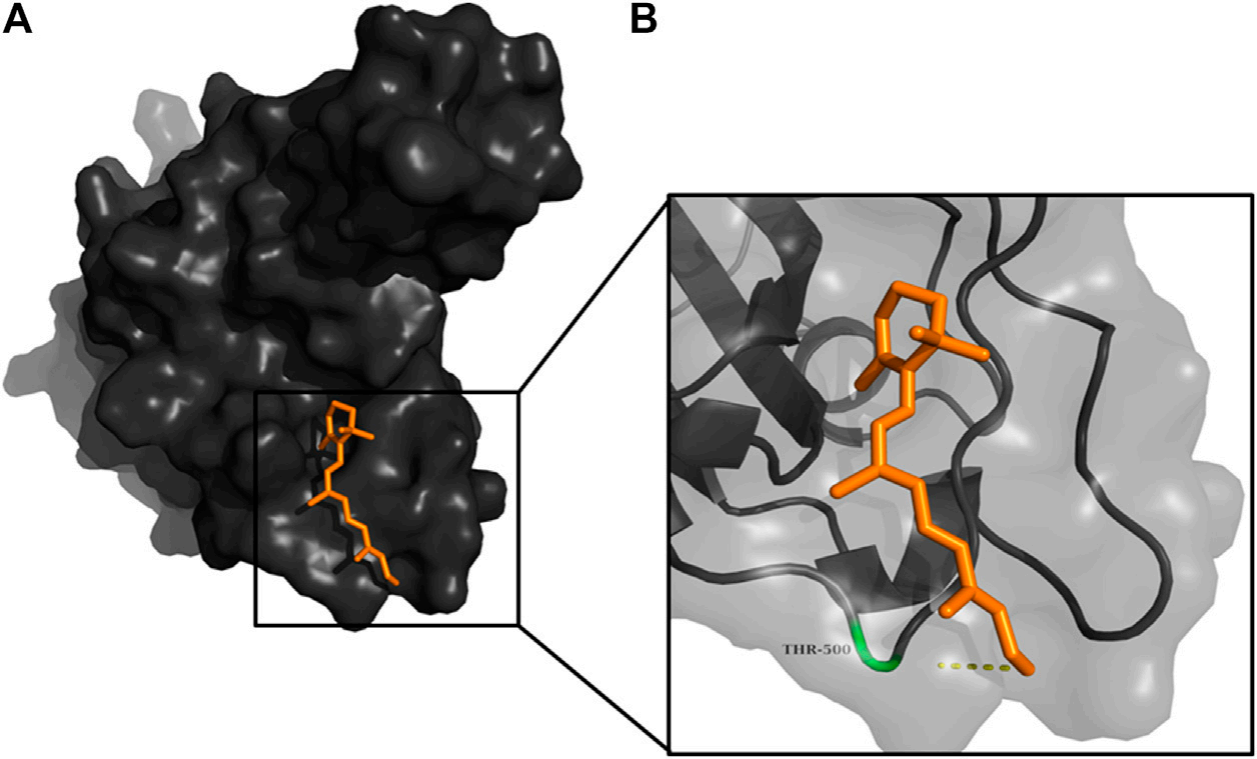
**(A)** The binding pose of retinal (orange) in RBD of S1 protein. **(B)** The hydrogen bond (yellow) formed between retinal and the interacting residue, Thr500, is also shown.

**FIGURE 9:**
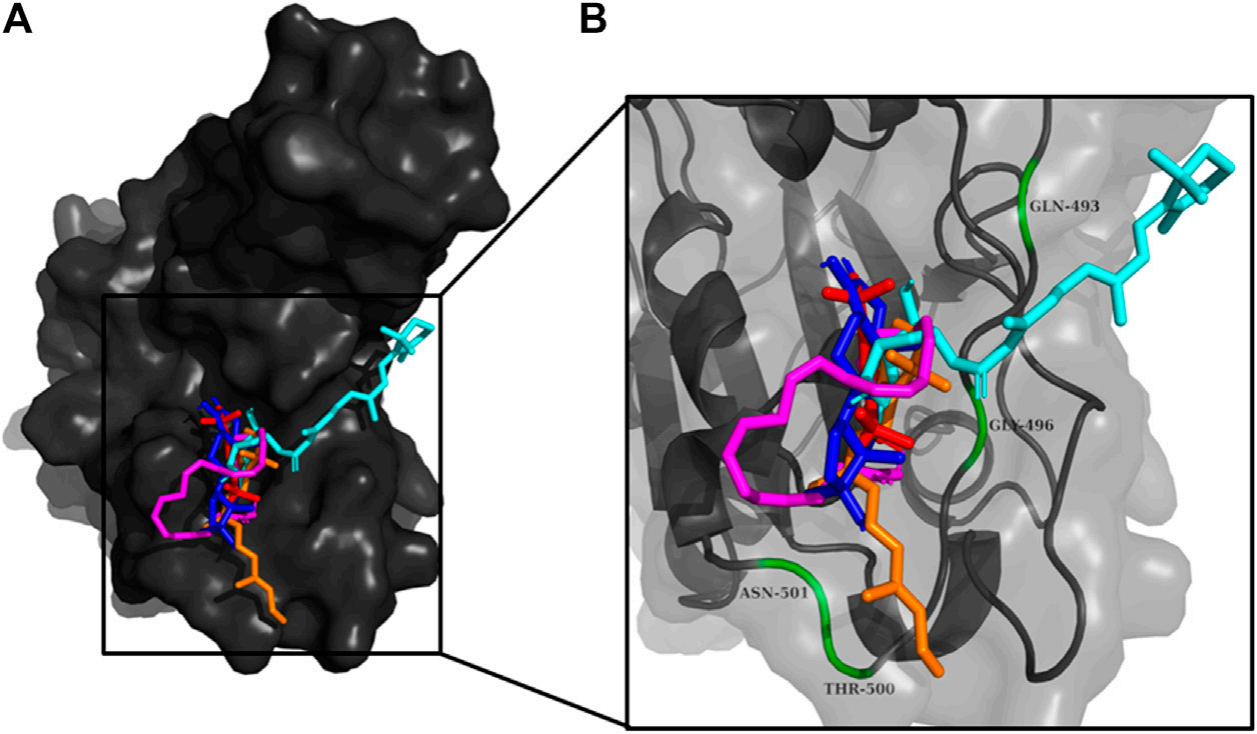
**(A)** The binding poses of the selected compounds from U. intestinalisextract in one of the active binding pockets of RBD of S1 protein. **(B)** The pocket residues interacting with the compounds are highlighted in green.

Hydroxychloroquine, the control ligand, showed a binding affinity of −5.7 kcal.mol^−1^ with the optimized structure of RBD. Twenty-one (48.84%) compounds had binding energies ranging from −4.0 kcal.mol^−1^ to −4.8 kcal.mol^−1^. Out of the 43 compounds, only 16 were considered for studying their molecular interaction (Tables 2, 3). Interaction analysis revealed that furfural had three hydrogen bonds interacting with Arg454, Ser469, and Glu471, but its binding energy was -3.8 kcal mol^−1^. Considering hydrophobic interactions, icosapent interacted with Arg403, Tyr453, Tyr495, Phe497, and Tyr505. The binding energy of this molecule was −4.8 kcal.mol^−1^. Out of these 16 compounds, only the best five compounds (2,4-DtBP, doconexent, DTD, RBGUL, and retinal) were considered for C-DFT, drug-likeliness studies using DruLiTo, and ADMET properties using pkCSM. The criteria used for this selection was mainly their relative lower binding energy. The conformations were visualized using PyMOL software and depicted in Figures 4–9.

**TABLE 2:** List of selected compounds identified from *Ui* extract with their two-dimensional chemical structures.

Compound	Structure	Compound	Structure
2,4-DtBP	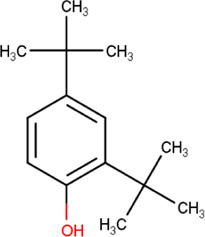	DPPP	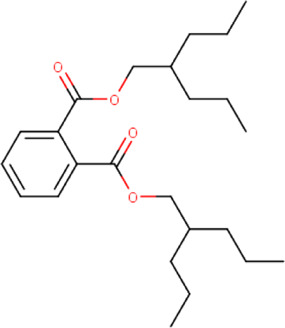
3-DOCH	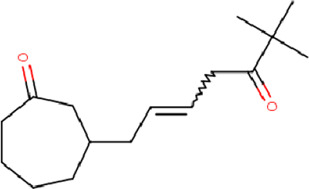	DTD	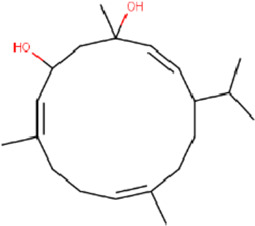
Azulene	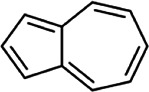	Furfural	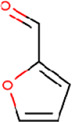
CMBA	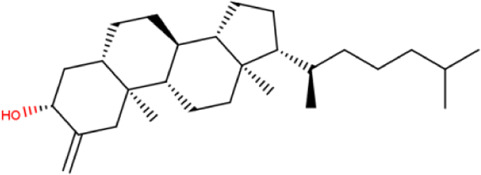	HIP	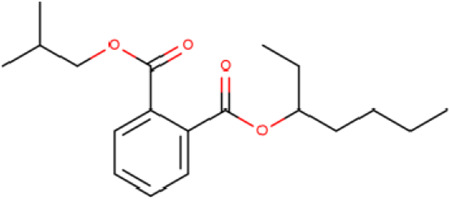
Cyclosativene	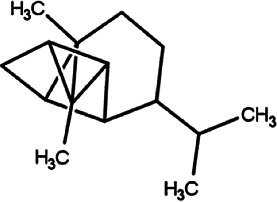	Icosapent	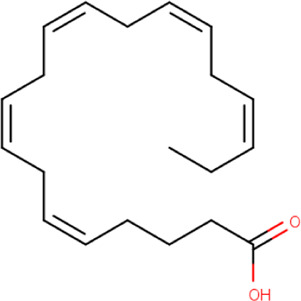
Damascone	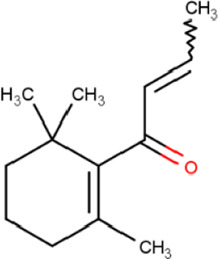	Oxymesterone	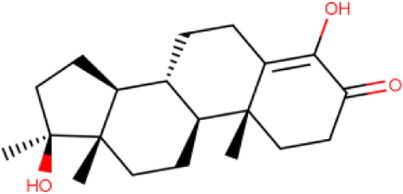
Dihydroactinolide	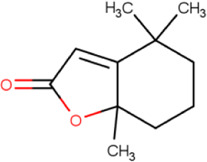	RBGUL	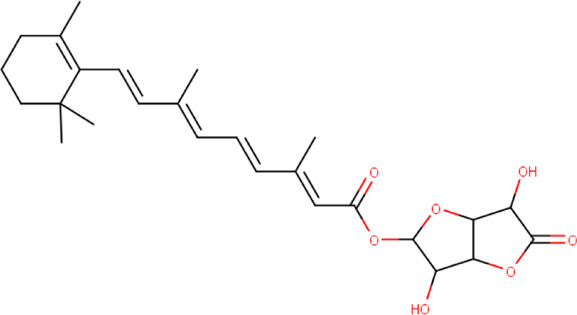
Doconexent	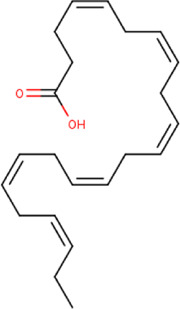	Retinal	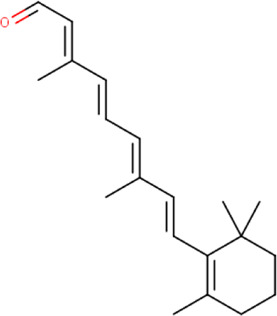

**TABLE 3:** **|** The binding affinities of selected phytochemicals from *Ui* extract on *SARS-CoV-2* spike RBD with the interacting amino acid residues contributing towards hydrogen bonds and hydrophobic interactions. The top five high scoring compounds have been highlighted (bold).

Compound	Binding affinity (kcal.mol^−1^)	Hydrogen bond interactions	Hydrophobic (pi) interactions
**2,4-DtBP**	**−5.3**	**Gly496, Asn501**	**Arg403, Tyr505**
3-DOCH	−5.3	−	Tyr505
Azulene	−5.1	−	Arg403, Tyr505
CMBA	−6.4	−	Tyr505
Cyclosativene	−5.3	−	−
Damascone	−5.2	−	Tyr505
Dihydroactinolide	-5.3	−	−
**Doconexent**	−**5.0**	**Gly496, Asn501**	**Arg403, Tyr495, Phe497, Tyr505**
DPPP	−4.8	Asn501	Tyr449, Tyr505
**DTD**	−**6.0**	**Gly496**	−
Furfural	−3.8	Arg454, Ser469, Glu471	Arg457, Lys458, Glu471
HIP	−5.2	−	Tyr505
Icosapent	−4.8	Gln498	Arg403, Tyr453, Tyr495, Phe497, Tyr505
Oxymesterone	−6.7	−	Tyr505
**RBGUL**	−**7.0**	**Gln493**	**Phe490**
**Retinal**	−**5.9**	**Thr500**	−

### Estimated Descriptors of Conceptual DFT

The molecular descriptors were calculated after optimization, based on the FMO theory (Table 4). The total energy of the compounds is the total electron energy of the ground state. Lower the total energy, higher is their stability. RBGUL displayed the lowest total energy with value −41.84 × 10^3^ eV. Molecular orbital energies such as HOMO energy (E_HOMO_) and LUMO energy (E_LUMO_) were calculated and analyzed (Table 5). Retinal showed the least energy gap with an energy difference of 3.04 eV. The energy gap of RBGUL (ΔE = 3.20 eV) was also found to be close enough to that of retinal. The maximum D_p_ was also shown by retinal (D_p_ = 6.33 Debye units). Considering derived descriptors, the most electronegative compound in the selected list was retinal (χ = 3.82). The electronegativity of RBGUL (χ = 3.67) was found to be highly similar to that of retinal. Absolute hardness and Global softness are criterions of overall stability of the system and also they are supporting parameters of electronegativity. In our study Retinal and RBGUL showed acceptable values of absolute hardness, 1.52 and 1.60 and softness, 0.33 and 0.31, respectively. Chemical potential of compounds is the negative value of electronegativity values, which is also an indication of high chemical activity. Therefore in this case too, retinal and RGBUL exhibited high chemical potential. High electrophilicity of retinal (4.80) and RBGUL (4.21) suggests their elevated likeliness to accept electrons. According to the above findings, RBGUL, and retinal were considered good inhibitors of S_1_ RBD of *SARS-CoV-2*.

**TABLE 4:** **|** Statistics of the conceptual DFT-global reactivity descriptors and their derivatives of the best phytochemicals.

Compound	Total energy, E_γ_×10^3^(eV)	Dipole moment(Debye)	E_LUMO_(eV)	E_HOMO_(eV)	Energy gap (ΔE)	Absolute hardness(η)	Global softness(σ)	Electro negativity (χ)	Chemical potential (μ)	Electro philicity index (ψ)
2,4-DtBP	−16.92	1.32	0.16	−5.68	5.85	2.92	0.17	2.76	-2.76	1.30
Doconexent	−27.43	1.04	0.17	−6.32	6.49	3.24	0.15	3.08	-3.08	1.46
DTD	−25.39	2.64	−0.03	−6.11	6.09	3.04	0.16	3.07	-3.07	1.55
RBGUL	−41.84	3.61	−2.07	−5.27	3.20	1.60	0.31	3.67	-3.67	4.21
Retinal	−23.24	6.33	−2.30	−5.34	3.04	1.52	0.33	3.82	-3.82	4.80

**TABLE 5:** Electron density maps of LUMO and HOMO of the top phytochemicals.

Compound	Optimized structure	LUMO	HOMO
2,4-DtBP	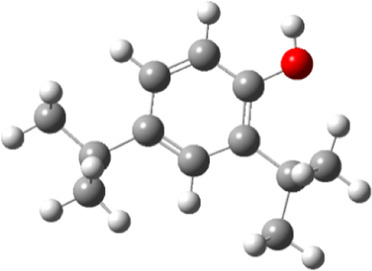	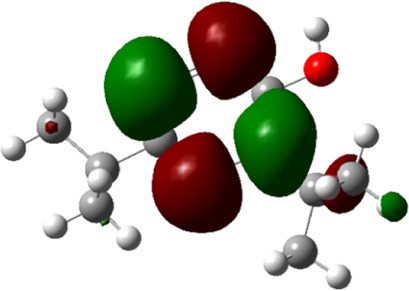	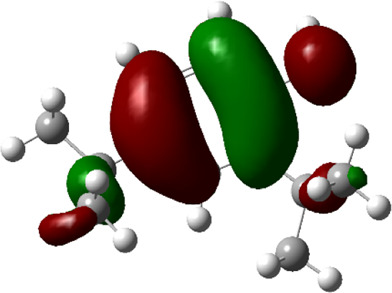
Doconexent	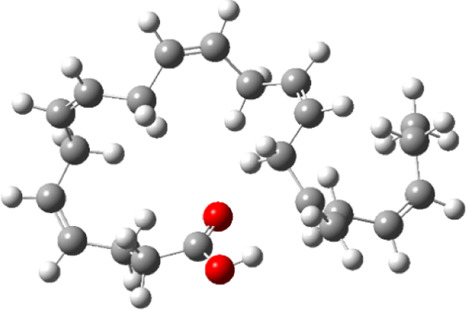	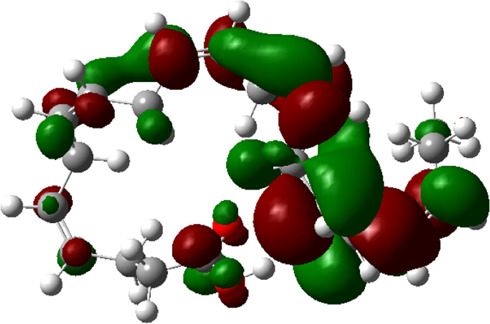	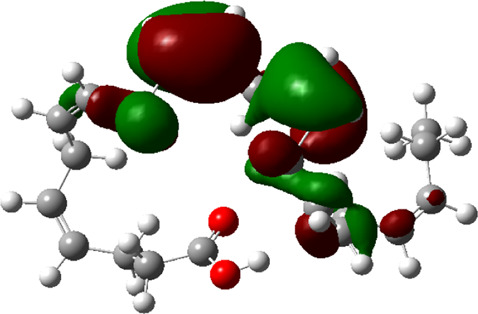
DTD	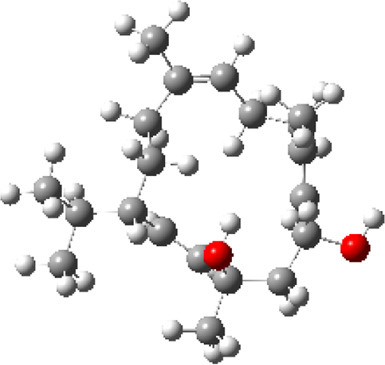	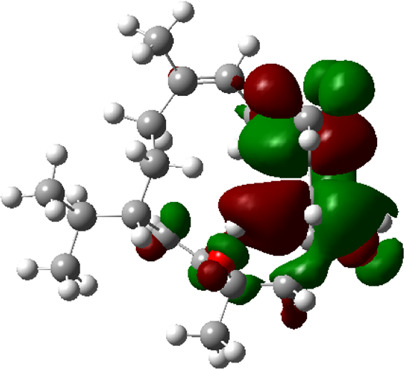	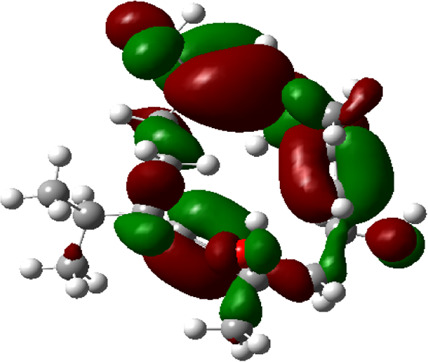
RBGUL	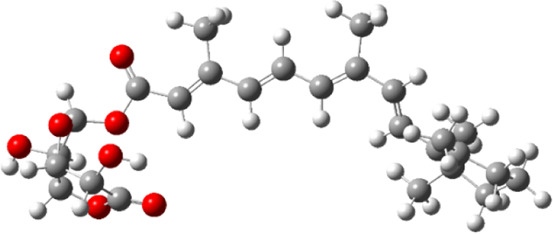	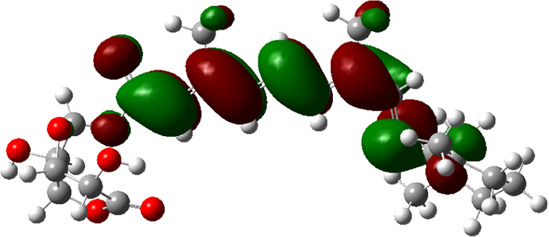	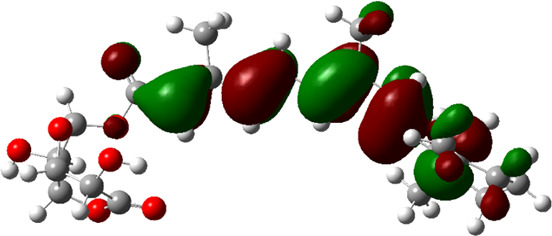
Retinal	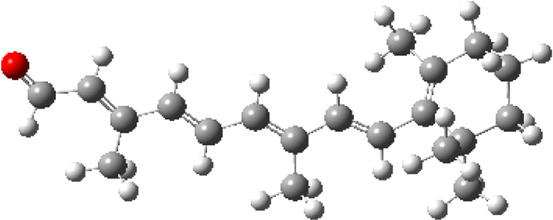	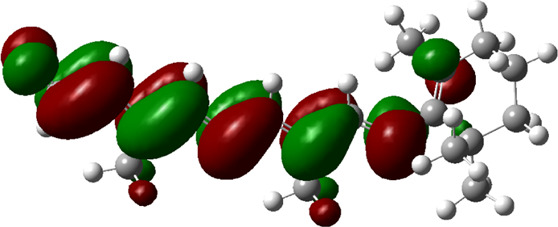	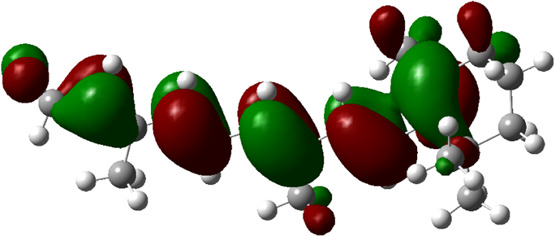

The red blobs represent the negative charge-dense regions and the green blobs represent the positive charge-dense regions of the molecule.

### Prediction of Pharmacokinetic Properties and Drug-Likeliness

The drug-likeliness prediction from DruLiTo and ADMET results from pkCSM are presented in Table 6. Evaluation of drug-likeliness showed that 2,4-DtBP satisfied and passed through the Lipinski’s RO5, Ghose, and Veber filters, whereas other ligands violated atleast one of the three parameters. Absorption properties revealed that all ligands were readily absorbed intestinally. 2,4-DtBP, doconexent, DTD, and retinal showed no interference with the P-glycoprotein system, however, RBGUL was found to be both a substrate and an inhibitor in the system. Skin permeability prediction showed that 2,4-DtBP was slightly permeable. Distribution properties showed that these compounds have tendencies to cross the blood-brain barrier (BBB) and central nervous system (CNS). Metabolic properties revealed that no ligand escaped the cytochrome P450 (CYP) system of the liver completely. Amongst the five selected ligands, DTD and RBGUL showed minimum interference with the system (acted as CYP2C19 inhibitor and CYP3A4 substrate, respectively). Considering excretion and toxicity properties, no ligand acted as renal OCT2 substrate, and human *ether-à-go-go*-related gene (*hERG*)-I protein inhibitors. The compounds passed the Ames toxicity test, indicating their inability to be a mutagen and thus a carcinogen. However, hepatotoxicity was predicted with doconexent, RBGUL, and retinal. Except for RBGUL, all other selected ligands showed skin sensitization too.

**TABLE 6:** Molecular and ADMET properties of the selected ligands by DruLiTo and pkCSM online tool.

Property	2,4-DtBP	Doconexent	DTD	RBGUL	Retinal
**Drug-likeliness**					
Molecular mass (Da)	206.17	328.24	306.26	458.23	284.21
Log*P*	4.279	8.833	5.228	5.067	6.335
No. of H-bond acceptors	1	2	2	7	1
No. of H-bond donors	1	1	2	2	0
Atom molar refractivity	69.37	111.27	96.24	125.44	95.7
No. of atoms	37	56	56	67	49
TPSA (Å^2^)	20.23	37.3	40.46	102.29	17.07
No. of rotatable bonds	2	14	1	7	5
Violation of Lipinski’s Rule	No	Yes	Yes	Yes	Yes
Pass through Ghose Filter	Yes	No	Yes	Yes	No
Pass through Veber Filter	Yes	No	Yes	Yes	Yes
**Absorption**					
log*S* (log mol/L)	−3.924	−6.098	−4.709	−4.62	−6.888
Caco2 permeability (logP app in 10^−6^ cm/s)	1.666	1.145	1.636	0.759	1.53
Human intestinal absorption (% absorbed)	92.034	92.98	92.426	72.172	94.747
Log*Kp*	−2.301	−2.731	−2.779	−2.897	−2.491
P-glycoprotein substrate	No	No	No	Yes	No
P-glycoprotein-I inhibitor	No	No	No	Yes	No
P-glycoprotein-II inhibitor	No	No	No	Yes	No
**Distribution**					
Human VDss (log L/kg)	0.611	−0.709	0.11	0.017	0.506
Human fraction-unbound (Fu)	0.044	0.001	0.256	0.211	0.04
Log*BB*	0.478	−0.203	0.4	−0.088	0.664
Log*PS*	−0.848	−1.169	−2.865	−3.051	−1.863
**Metabolism**					
CYP2D6 substrate	No	No	No	No	No
CYP3A4 substrate	Yes	Yes	No	Yes	Yes
CYP1A2 inhibitor	Yes	Yes	No	No	Yes
CYP2C19 inhibitor	No	No	Yes	No	No
CYP2C9 inhibitor	No	No	No	No	No
CYP2D6 inhibitor	No	No	No	No	No
CYP3A4 inhibitor	No	No	No	No	No
**Excretion**					
Total clearance (log mL/min/kg)	0.759	2.264	1.376	0.861	1.563
Renal OCT2 substrate	No	No	No	No	No
**Toxicity**					
Ames toxicity	No	No	No	No	No
Human MRTD (log mg/kg/day)	0.42	−0.98	0.483	−0.142	−0.341
*hERG-I* protein inhibitor	No	No	No	No	No
*hERG-II* protein inhibitor	No	No	No	No	Yes
ORAT-LD50 (mol/kg)	2.351	1.459	1.673	1.913	1.564
ORCT-LOAEL (log mg/kg_bw/day)	1.696	3.208	2.002	2.579	1.065
Hepatotoxicity	No	Yes	No	Yes	Yes
Skin sensitization	Yes	Yes	Yes	No	Yes
*T. pyriformis*toxicity (log μg/L)	1.572	0.451	1.348	0.285	1.515
Minnow toxicity (log mM)	0.006	−1.765	0.528	0.373	−0.56

log*P*: Octanol-water partition coefficient; TPSA: Total polar surface area; log*S*: measure of water solubility; log*Kp*: measure of skin permeability; VDss: volume of distribution; log*BB*: measure of BBB permeability; log*PS*: measure of CNS permeability; OCT2: organic cation transporter 2; ORAT-LD50: oral rat acute toxicity-lethal dose 50; ORCT-LOAEL: oral rat chronic toxicity-lowest dose causing observed adverse effects.

## Discussion

Medicine has started to change from completely “synthetic” to “semi-herbal” in the last couple of decades. Due to the lack of effective treatment and management strategies to treat COVID-19, alternative therapies are being explored. Conventional drug development process involves elaborate and time-consuming protocols, and they seldom produce drugs on demand. To increase the complexity, the causative agent, *SARS-CoV-2*, is a virus with high mutability and variable reproduction number (Rahman et al., 2020) that is slightly greater than its pathological cousins, *SARS-CoV* and *MERS-CoV* (Liu et al., 2020). Due to these facts, it is challenging to develop drugs against this virus presently. However, drugs could be developed against conserved regions of its genome or proteins encoded from these regions, such as spike glycoprotein or main protease, and intense research is being conducted world-wide, for the same. Drug repurposing is the most accepted strategy considered in this approach. Using *in silico* techniques, commercially available drugs are docked with a target protein, and the screened drug could be made available for patients within a much shorter period because the clinical profile of the drug has been already established. Some drugs repurposed against *SARS-CoV-2* were Remdesivir, Favipiravir, Ribavirin, Lopinavir, Ritonavir, Darunavir, Tocilizumab, type I and type II interferons, chloroquine, hydroxychloroquine, arbidiol and statins (Singh et al., 2020). Though it is a fast-paced approach, *in vitro* and *in vivo* studies are required to fully understand its mechanism in the human body, especially when the stakes of comorbid symptoms are high with this disease.

The undesirable side-effects of synthetic drugs has attracted researchers, and scientists towards developing plant-based medicines. Various compounds obtained fromt extracts of plants that belong to families such as *Lamiaceae*, *Fabaceae*, *Geraniaceae*, *Rosaceae*, *Asteraceae*, *Rutaceae* and *Malvaceae*have been reported to exhibit antiviral activity against *SARS-CoV-2* and certain other viruses too (Drevinskas et al., 2018; Denaro et al., 2020; Siddiqui et al., 2020). The top compounds identified as potent antivirals in our study have been previously reported to have exhibited a wide array of functions. 2,4-DtBP is a lipophilic phenol found mostly in higher plants. The phenol and its analogs were reported to have anti-oxidant, anti-inflammatory, anti-cancer, and anti-microbial properties. Considering their anti-viral activities, they reduced the growth of Coxsackievirus B-3 and Herpes Virus type-2 (Zhao et al., 2020). Our study revealed that 2,4-DtBP binds to S_1_ RBD of *SARS-CoV-2* with a binding energy of −5.3 kcal.mol^−1^, and interacted with Gly496 and Asn501 by hydrogen bonds and Arg403 and Tyr505, hydrophobically. Doconexent is a fatty acid which is rich in docosahexaenoic acid (DHA), is a compound with high anti inflammatory properties which is commercially produced from certain microalgae (Milledge, 2011). It has been repurposed to treat cancer and COVID-19 (Li et al., 2020; Singhal et al., 2020; Stanly et al., 2020). Retinal is a vitamin A aldehyde in the most absorbable form. Many studies have pointed the role of vitamins which include retinal, in managing COVID-19 (Michele et al., 2020; Morais et al., 2020; Gröber and Holick, 2021). DTD is a macrocyclicditerpene, primarily isolated from the Tobacco plant (*Nicotianatabacum*). It was found to be a major constituent in the oil extract from the aerial parts of Hercules’ all-heal (*Opopanaxchironium*) (Maggio et al., 2013) and has a structural similarity with cembrene (Roberts and Rowland, 1962). Though DTD was not studied for its clinical properties, it was found that cembrenoid derivatives showed anti-cancer properties *in vitro* (Jassbi et al., 2017). With a binding affinity of −6.0 kcal.mol^−1^ against *SARS-CoV-2*, it proved to be a good inhibitor of the virus. RBGUL has similar properties to retinoic acid, and retinol. It was proposed to be a valuable therapeutic compound for the treatment of dermatological conditions and certain cancers, and also a dose-dependent teratogen (Barua, 1997). In our study, RBGUL was found to be the best inhibitor of *SARS-CoV-2*, compared to the other compounds with good binding affinity to the virus (−7.0 kcal.mol^−1^).

*In silico* techniques occupy a prominent role in early drug discovery process. A quantitative computational study of the interaction between a particular protein target and a set of ligands, provides a fair idea as to which of the ligands may have an effect on the protein *in vitro*. Screening a large number of compounds against a particular target to narrow down the number of compounds to be tested *in vitro* is easily achievable by bioinformatics techniques. Molecular docking aids in assessing and visualizing the interactions between the ligands and protein. Similarly, the C-DFT study performed by calculating global molecular descriptors based on DFT provides a quantum level understanding of the ligands and helps to construct the relationship between their electronic properties and biological activity. It can also be used to understand the quantitative structure-activity relationship and perform pharmacophore modeling to design effective drugs out of the existing, according to the target. RBGUL and retinal show similar electron density in the orbitals except that the structures look inverted, suggesting that the inhibitory action of both compounds may be similar. They were also considered as highly active compounds as they showed low ΔE, which helps in an easy transition from HOMO to LUMO. Comparing the results of docking and C-DFT, the compounds with higher electronegativity showed better activity. Thus it can be comprehended that smaller ΔE, high D_p,_ and low electronegativity are essential for the inhibitory effect of a molecule. However, compared to RBGUL, retinal had more disadvantages based on the pharmacokinetic predictions. Besides RBGUL, 2,4-DtBP is also a potential candidate against RBD of *SARS-CoV-2*, considering its less adverse effects. That being said, the most recommended inhibitors against RBD would be 2,4-DtBP and RBGUL. More studies on these phytochemicals can reveal their efficacy, thus validating the results of this experiment.

## Conclusion

Phytochemicals obtained from *Ui* extract were docked with the *SARS-CoV-2* RBD to ascertain if it exhibited antiviral activity, and also to screen for the compounds that are responsible for the activity. Through this study, we conclude that RBGUL, 2,4-DtBP and Retinal could be used as potent inhibitors against the RBD of coronavirus based on the molecular docking, C-DFT and ADMET studies. However, further studies involving *in vitro* and *in vivo* testing is essential to confirm the antiviral efficiency of the compounds against *SARS-CoV-2*.

## Data Availability

The raw data supporting the conclusions of this article will be made available by the authors, without undue reservation.
